# Syphilis seroprevalence and associated factors: A cross-sectional study in formal female sex workers in a province of Peru

**DOI:** 10.1097/MD.0000000000037774

**Published:** 2024-04-12

**Authors:** Vanessa Huamaní-Chavez, Lenin Rueda-Torres, Luis Ormeño-Delgado, Jair Li, Jaime Rosales-Rimache

**Affiliations:** a Universidad San Pedro, Lima, Peru; b Universidad César Vallejo, Trujillo, Peru; c Universidad Científica del Sur, Lima, Peru; d Universidad Nacional Federico Villarreal, Lima, Perú; e Universidad Privada Norbert Wiener, Lima, Peru.

**Keywords:** female sex worker, Peru, risk factor, syphilis

## Abstract

Syphilis is a sexually transmitted infection (STI) considered a public health problem that affects vulnerable and at-risk populations, such as sex workers. We designed a retrospective cross-sectional study based on the review of health evaluation records of sex workers who attended consultations to obtain comprehensive health cards at a Health Center in the Province of San Vicente de Cañete in Peru during the year 2020. We obtained sociodemographic and employment information and the RPR (rapid plasma reagin) test results to diagnose syphilis. We evaluated 220 records of sex workers with a mean age of 27.9 ± 6.9 years and the initiation of sexual relations of 16.0 ± 1.6 years, while the accumulated time they had as sex workers was 2.9 ± 2.4 years. 85.9% of those evaluated reported being heterosexual. The prevalence of syphilis was 7.3% (CI95: 4.2%–11.5%). Being a homosexual sex worker was significantly associated with syphilis (OR: 19.6; 95% CI: 4.8–80.0) compared to heterosexuals. The prevalence of syphilis presented a value similar to that reported in other Latin American and national studies, and it is evident that it is a health problem among sex workers.

## 1. Introduction

Syphilis is a chronic systemic infectious disease transmitted mainly by sexual contact or vertical transmission during pregnancy.^[1]^ It is caused by *Treponema pallidum*, a pathogenic spirochete bacterium unique to humans and endemic in low-income countries.^[2]^ The importance for individual and public health lies in the lack of early diagnosis and treatment of patients who can progress to chronic latent stages with severe neurological and cardiovascular complications.^[3]^ On the other hand, pregnant women are associated with the risk of miscarriages, prematurity, and low birth weight, among other perinatal events of long-term morbidity for the neonate.^[4]^

Annually, more than 9 million new cases of syphilis in adults are reported worldwide,^[5]^ of which almost a third are registered in Latin American and Caribbean countries.^[6]^ In Peru, the general prevalence of syphilis is estimated at 0.45%, which doubles in female sex workers (FSW).^[7]^ In contrast, the incidence of congenital syphilis reaches 4.70 per 1000 live births, one of the highest rates in Latin America.^[8]^ The most important risk factors are associated with risky sexual behavior practices, with the main transmission sources being men who have sex with men, male and female sex workers, clients of sex workers, and the transgender population.^[9]^

FSWs are one of the high-risk groups for contracting sexually transmitted infections (STI), such as syphilis, due to conditions associated with their occupation, including multiple sexual partners, inconsistent condom use, and coinfections with other STIs.^[10,11]^ Although this voluntary work activity is regulated in Peru, the marginalization and social stigma towards the FSW population promote informality and exploitation under precarious conditions on the streets and brothels. This situation increases risk behavior, violence, and rejection of STI screening.^[12]^ Added to this, in recent years, the migratory flow of more than 1 million Venezuelans in poverty^[13]^ and the COVID-19 pandemic^[14]^ have exacerbated the recurrence of women to sex work, intensifying the market with more significant barriers in seeking health care and low adherence to health programs.^[15,16]^

The STI epidemiological surveillance standard in Peru includes FSW within the risk groups and pregnant women, prisoners, and injecting drug users.^[17]^ However, given the passive search for cases, significant under-registration at the national level is likely, especially in provinces, where working conditions and primary care facilities are limited.^[18,19]^

Laboratory tests are essential for diagnosing syphilis, complementary to the clinical manifestations. It is recommended to use both treponemal and non-treponemal serological tests to achieve good sensitivity and specificity in identifying asymptomatic infections.^[20]^ According to the Peruvian clinical guide, screening to identify probable cases of syphilis infection is through non-treponemal tests, RPR (rapid plasma reagin) or VDLR (Venereal Disease Research Laboratory), with the antibody titer equal to or greater than 8 dilutions being the indicator of active disease. For confirmation, there is support from reference regional laboratories or the National Institute of Health that applies treponemal tests: TPHA (*Treponema pallidum* Hemagglutination assay), TP-PA (*Treponema pallidum* particle agglutination), or FTA-Abs (fluorescent treponemal antibody absorption).^[17]^

Our study aimed to determine the seroprevalence of syphilis and its associated factors in FSW in the province of Cañete in Lima during the year 2020, based on the review of medical history records generated in the health evaluation at a Health Center. Our study is relevant to improving primary and secondary prevention strategies in this risk group, whose socioeconomic and cultural characteristics place them as a vulnerable population.

## 2. Methods

### 2.1. Study design and participants

We designed a retrospective cross-sectional study by reviewing FSW medical records. We collected information from sentinel surveillance records in FSWs that were attended during February and March 2020 at the San Vicente de Cañete health center, a small province on the central coast located 111 km from Lima, the capital of Peru. The medical care that the workers received corresponds to the periodic general control (not yet regulated) necessary to obtain a health card. This requirement allows them to continue working within brothels authorized by the regional authority. We included records of FSWs of legal age who were treated as part of obtaining a health card. We excluded records that did not have information on laboratory test results for syphilis and incomplete or implausible data.

### 2.2. Techniques and instruments

Information was collected from the medical records in a form for exhaustive collection of epidemiological data, such as age, nationality, ethnicity, sexual orientation, initiation of sexual relations, marital status, level of education, number of clients seen per day, history of STI, and laboratory test results for syphilis. The laboratory of the San Vicente de Cañete Health Center used the qualitative determination of RPR (Spinreact, Spain) in serum samples from venous blood obtained by venipuncture. The RPR was performed according to the manufacturer’s established procedure. The reading was performed by visual macroscopic examination, and the presence of large or medium-sized aggregates after 8 minutes of rotation of the reagent with the sample was considered a reactive test. The results with the reaction of small aggregates were worked in duplicate, and the result’s recurrence was considered reactive. Only the absence of additions to the visual observation was considered as nonreactive evidence. The execution of the test was subject to compliance in the use and evaluation of internal controls (negative and positive). It should be noted that the RPR test used indicates a diagnostic sensitivity and specificity of 100%, according to the manufacturing insert. The final information was transcribed into a double-typing spreadsheet, with subsequent verification and quality control of the data.

### 2.3. Statistical analysis

We presented the descriptive characteristics of the study population according to their measurement scale. The prevalence of syphilis was presented as a percentage with a 95% confidence interval. Likewise, this was estimated according to each secondary variable considered in the study, and in a complementary manner, their proportions were compared using Pearson’s chi-square test. Identifying factors associated with syphilis was evaluated in a logistic regression model, with calculation of the Odds Ratio and its 95% confidence interval. We carried out the selection of variables according to epidemiological criteria. We consider a probability <0.05 as a significant value. Statistical analysis was performed in Stata version 17 (StataCorp. LCC College Station, TX, USA).

### 2.4. Ethical aspects

The San Pedro de Chimbote University approved the study with Official Letter No. 187-2020-FCS-EPTM-USP. In addition, we obtained permission from the San Vicente de Cañete Health Center, and the study did not require obtaining informed consent due to the retrospective design. The database was anonymized to guarantee the confidentiality of information, and access was only for the study researchers.

## 3. Results

We evaluated 232 records from FSW treated at the San Vicente de Cañete Health Center in 2022; however, we excluded 12 records due to a lack of results for the RPR test. The mean age was 27.9 years, the start of sexual relations was 16.0 years, and their accumulated time as FSW was 2.9 years. On the other hand, the median number of clients served per week was 12. 68.6% of the FSW reported being single, while 25.5% coupled. The majority of education was between primary and secondary, with 46.8 and 45.5%, respectively.

Regarding sexual orientation, 85.9% of those evaluated reported being heterosexual. More than 30% of those evaluated had foreign nationality, and 14.1% indicated having had STI. Among the latter, syphilis, candidiasis, and trichomoniasis stand out. One case of genital herpes and another of HIV infection were found. The prevalence of reactive results for syphilis in FSW was 7.27%, with a 95% confidence interval of 4.21% to 11.54% (Table 1).

**Table 1:** Descriptive characteristics of the study population (n = 220).

Characteristic	n (%)	95% CI
Age (yrs)	27.9 ± 6.9 (mín.: 18 y máx.: 52)^a^	
Age of initiation of sexual relations (yrs)	16.0 ± 1.6 (mín.: 7 y máx.: 20)^a^	
Time as a sex worker (yrs)	2.9 ± 2.4 (mín.: 0.1 y máx.: 18)^a^	
Number of clients served per day	12 (9–17)^b^	
Civil status		
Single woman	151 (68.6)	62.2–74.5
Married	12 (5.5)	3.1–9.4
Cohabitant	56 (25.5)	20.1–31.7
Widowed/Divorced	1 (0.4)	0.1–3.2
Degree of instruction		
Primary	103 (46.9)	40.3–53.5
Secondary	100 (45.5)	39.0–52.1
Superior	17 (7.7)	4.8–12.1
Sexual orientation		
Heterosexual	189 (85.9)	80.6–89.9
Homosexual	31 (14.1)	10.1–19.4
Ethnicity		
White	11 (5.0)	2.8–8.8
Black	6 (2.7)	1.2–6.0
Mixed race	203 (92.3)	87.9–95.2
Nationality		
Peruvian	147 (66.8)	60.3–72.8
Foreigner	73 (33.2)	27.2–39.7
History of STI		
No	189 (85.9)	80.6–89.9
Yes	31 (14.1)	10.1–19.4
Syphilis		
No	204 (92.7)	88.4–95.5
Yes	16 (7.3)	4.5–11.6

a Mean and standard deviation.

b Median and interquartile range.

The variables independently associated with the syphilis-reactive outcome were age, age of initiation of sexual relations, time as FSW, sexual orientation, nationality, and history of STIs. An older age, a higher age of initiation of sexual relations, and a longer time as FSW are observed in those with a reactive result for syphilis compared to FSWs with a nonreactive result for syphilis. In homosexual workers, a higher frequency of reactivity to syphilis is recorded compared to heterosexual workers. There were no foreigners with syphilis, and those with a history of STIs had a slightly higher frequency than those without a history of STIs (Table 2).

**Table 2:** Factors independently associated with syphilis in bivariate analysis (n = 220).

Characteristic	Syphilis, n (%)		*P*-value
	No	Si	
Age (yrs)	27.4 ± 6.3	34.4 ± 10.1	<.001^a^
Age of initiation of sexual relations (yrs)	15.9 ± 1.6	17.1 ± 1.4	.009^a^
Time as a sex worker (yrs)	2 (1–4)	3 (2–5)	.011^b^
Number of clients served per day	12 (9–18)	6.5 (4.5–12.5)	.279^b^
Civil status			.632^c^
Single woman	138 (91.4)	13 (8.6)	
Married	12 (100.0)	0 (0.0)	
Cohabitant	53 (96.6)	3 (5.4)	
Widowed/Divorced	1 (100.0)	0 (0.0)	
Degree of instruction			.099^c^
Primary	94 (91.3)	9 (8.7)	
Secondary	96 (96.0)	4 (4.0)	
Superior	14 (82.4)	3 (19.7)	
Sexual orientation			<.001^d^
Heterosexual	184 (97.4)	5 (2.7)	
Homosexual	20 (64.5)	11 (35.5)	
Ethnicity			.486^c^
White	11 (100.0)	0 (0.0)	
Black	6 (100.0)	0 (0.0)	
Mixed race	187 (92.1)	16 (7.9)	
Nationality			.008^d^
Peruvian	131 (89.1)	16 (10.9)	
Foreigner	73 (100.0)	0 (0.0)	
History of STI			<.001^d^
No	189 (100.0)	0 (0.0)	
Yes	15 (48.4)	16 (51.6)	

a Two-tailed student *t* test.

b Non-parametric Mann–Whitney test.

c Pearson’s chi-squared test.

d Pearson’s chi-squared test with Yates’s correction for continuity.

The multivariate analysis identified that homosexual workers had almost 20 times the chance of having syphilis compared to heterosexual workers (Table 3).

**Table 3:** Factors independently associated with syphilis in multivariate analysis (n = 220).

Characteristic	Bivariate analysis^a^		Multivariate analysis^a^	
	OR (95% CI)	*P*-value	OR (95% CI)	*P*-value
Age (yrs)	1.12 (1.05–1.20)	<.001	1.03 (0.95–1.12)	.488
Age of initiation of sexual relations (yrs)	1.69 (1.15–2.50)	.008	1.24 (0.83–1.87)	.293
Time as a sex worker (yrs)	1.10 (0.92–1.31)	.277	1.20 (0.95–1.52)	.127
Sexual orientation				
Heterosexual	Reference		Reference	
Homosexual	20.24 (6.39–64.14)	<.001	19.60 (4.80–80.04)	<.001

a Logistic Regression.

## 4. Discussion

Our results show a 7.3% seroprevalence of syphilis in FSW. The prevalence of FSW is variable between cities and populations; analyzed values from 32 countries show an average of 10% of active syphilis with a wide range between 5.8% and 30.0%.^[21]^ To our knowledge, there is no epidemiological data on syphilis in FSW in provinces in Peru. A systematic review reported syphilis prevalence for Peru in risk groups of 2.2% to 4%,^[9]^ which is exceeded in this study. When comparing our findings, it is more similar to prevalences reported by low-income countries in Latin America, such as the Dominican Republic (5.1%–11.1%),^[22]^ Guatemala (1.1%–11.8%)^[23]^ and El Salvador (2.7%–15.0%).^[24]^

Cañete is a small province on the north coast of the capital city, Lima, whose main economic activity is agriculture and has an intermediate poverty index depending on the location of total monetary poverty.^[25]^ These socioeconomic conditions lead to a lack of health care, knowledge, and prevention of unsafe practices that influence the appearance of syphilis in the population.^[26]^ For example, a study obtained a prevalence of syphilis of 8.59% (95% CI 5.78–12.59), identifying as risk factors the low percentage of condom use with a stable partner, high drug consumption, and low-risk perception.^[27]^

Although educational level is not an independent risk factor for syphilis infection, the level of education is one of the most essential variables in measuring socioeconomic status and its effects on the health status of a population. It has been observed that FSWs in brothels have a better level of knowledge about STIs, reducing risk behaviors and impacting the prevalence of syphilis. On the other hand, low educational quality is a constant problem in provincial communities and educational centers in Peru.^[28]^ A notable finding is that almost 80% of those evaluated reported having initiated sexual activity as minors. The evidence supports this association with the risk of contracting sexually transmitted infections. Other researchers have shown that early age at the beginning of sexual activity and work significantly increases the risk of STD.^[27]^

We observed that the number of clients served per day does not behave as a risk factor associated with syphilis; in fact, the highest prevalence was found in those who had fewer clients per day. However, it is essential to specify that the risk of infection does not necessarily increase due to the number of contacts with clients but rather due to inadequate practices, such as the lack of use of condoms, lubricants, and an increase in the frequency of anal sex. Anal intercourse increases the risk of STIs due to the fragility of the tissue, sensitive to injury, which gives bacteria and viruses access to the bloodstream.^[1]^ In this sense, the group at most significant risk for syphilis is men who have sex with men.^[29]^

The adjusted analysis shows a strong association of homosexual orientation with the reactive syphilis test (OR = 19.6, 95% CI 4.8–80.04). This finding is relevant, although little studied in our region. It has previously been shown that lesbian women who report having sexual relations with other women can contract and increase the risk of STDs.^[30]^ The plausible biological explanation for this event would be based on the sexual behaviors of said practice; genital contact, insertive sex, and oral-vaginal/vulval contact can increase the transmission of viruses such as herpes, HIV, and syphilis.^[31]^ On the other hand, it is also maintained that sexual contact between women during the menstrual period further increases the probability of contagion by having contact with blood.^[32]^

In the case of FSW, it can be suspected that in addition to the routine job of working with male clients, these workers would maintain contact with each other and others, closing a circle of high-risk of transmission. Women who have sex with both genders (WSB) are reported to be more likely to engage in transactional sex, drug use, and excessive alcohol consumption compared to women who have sex with women (WSW) and women who have sex with men (WSM).^[33]^ Some studies show that the risk of syphilis in WSB is equal to or slightly higher than in WSW and WSM,^[33,34]^ although not to the magnitude of what was found in the present study.

On the other hand, the COVID-19 pandemic generated an economic recession and could increase the supply of informal sexual services, increasing risk behaviors and, therefore, STIs. This situation has already been evidenced in sex workers from other countries.^[35,36]^ Likewise, the infection and transmission of Treponema pallidum are related to social, economic, cultural, and behavioral factors that influence the appearance of syphilis in the population, especially among sex workers.

Given the study’s design, the limitations are conditioned by the information sources’ availability, integrity, and potential biases in record-keeping. No data on the attitudes, practices, and perceptions of sex with the FSW partner were recorded. On the other hand, the marker used was a non-treponemal serological test, which is not confirmatory and may err in latent cases or pre- and prozone effects; however, it is the method endorsed within the framework of national regulation for STI surveillance. in primary care.

## 5. Conclusion

Syphilis is a public health problem in risk groups such as sex workers. We found a medium to high seroprevalence of syphilis among sex workers in a province of Peru. Likewise, some important associated factors, among them, homosexual orientation, stand out, a finding that should be more explored in the current context. STIs not only threaten the most vulnerable marginalized populations, such as sex workers but can spread throughout society, mainly when associated with the sex trade. Controlling infection in at-risk populations also helps control the spread of disease. Therefore, it is crucial to implement and improve educational programs to present and discuss the risks involved in sexual practices. It is also crucial to guarantee screening through rapid syphilis tests and self-assessment possibilities to identify the infection timelily.

## Acknowledgments

We thank Universidad Continental for paying the APC.

## Author contributions

Conceptualization: Jaime Rosales-Rimache, Vanessa Huamaní-Chavez.

Data curation: Lenin Rueda-Torres, Luis Ormeño-Delgado.

Formal analysis: Vanessa Huamaní-Chavez, Lenin Rueda-Torres, Luis Ormeño-Delgado, Jair Li.

Investigation: Vanessa Huamaní-Chavez, Lenin Rueda-Torres, Jair Li.

Methodology: Vanessa Huamaní-Chavez.

Project administration: Vanessa Huamaní-Chavez.

Supervision: Jaime Rosales-Rimache.

Validation: Jaime Rosales-Rimache.

Visualization: Jair Li.

Writing – original draft: Vanessa Huamaní-Chavez, Lenin Rueda-Torres, Luis Ormeño-Delgado, Jair Li.

Writing – review & editing: Jaime Rosales-Rimache.
